# Infant sleep as a topic in healthcare guidance of parents, prenatally and the first 6 months after birth: a scoping review

**DOI:** 10.1186/s12913-022-08484-3

**Published:** 2022-09-08

**Authors:** Inger Pauline Landsem, Nina Bøhle Cheetham

**Affiliations:** 1 The Arctic University of Norway, The Health Research Faculty, Institute of Health and Caring Science, Hansine Hansens veg 18, 9019 Tromsø, Norway; 2 Child and Adolescent Department, University Hospital of North Norway, Sykehusvegen 38, 9019 Tromsø, Norway

**Keywords:** Infant sleep, Sleep safety, Place of sleep, Parental guidance, Sleep consolidation, Bedtime routines

## Abstract

**Background:**

This scoping review focuses on infant sleep-related factors and themes that are relevant when health practitioners provide preventive health services to expectant and new parents.

**Methods:**

A systematic literature search in CINAHL, PubMed, and PsycINFO, published in 2010 or later, identified 1661 records. The search was further narrowed to focus on sleep in healthy term-born infants until the second half of the first year of life. A blinded review by both authors covered 136 papers, of which 43 papers were reviewed in the full text. Finally, 38 articles were included in the data extraction.

**Results:**

The analysis process showed that the selected studies formed three main information categories: 11 studies thematised safe infant sleep issues, 10 studies described design and findings from sleep-related intervention studies, and 17 studies focused on different parent-child interactive aspects that may influence the quality and duration of infant sleep in the first six months of life.

The main finding is that knowledge about early infant sleep is very complex, and includes both child, parent, and environmental factors. Several studies have shown that the concepts and factors related to safe infant sleep also influence the development of healthy infant sleep patterns. Thus, these aspects are interwoven with each other and should be addressed together in communication with parents.

**Conclusions:**

Health practitioners with different professional backgrounds need to search for an agreement on when and how different aspects of sleep-related knowledge should be communicated to new and expectant parents to enable the design of national follow-up programs. Parents want coherent and personalized services regarding infant sleep issues that may allow them to choose sleeping arrangements, routines, and behaviors that fit in with their sociocultural attitudes and traditions. Many different sources and formats may be used to empower parents regarding infant sleep issues. Studies have described the use of group or individual meetings, videos, and written materials. The key issue is the importance of consistent and seamless knowledge-based services.

## Background

Establishing nighttime-sleep (NTS) in early infancy is challenging for many parents. Both professionals and parents have summarized these difficulties in recent Norwegian publications [1–3] and research has documented that many parents struggle to follow advices on safe infant sleep, despite regular campaigns to promote such information [4]. The importance of sufficient sleep is well known, and updated recommendations on sleep duration for groups of children aged five years or less were published by the World Health Organization (WHO) in 2019 [5]. Short sleep duration or interrupted sleep in infancy is associated with increased sleeping difficulties later in childhood [6], as well as risks of a range of health problems [7, 8]. Infant sleep is a complex phenomenon associated with factors as parental sleep and mental health [9], maternal sensitivity [10] and parental practices [11].

Most healthcare professionals know that the first six months of infancy are characterized by maturation and sleep consolidation [12, 13]. The term “sleep problems” is rarely used until the infant enters the second half of the first year [1]. Research has identified this early consolidation phase as important for determining whether sleep-related problems occur later in infancy or toddlerhood [14, 15].

Parent facilitation of infant sleep is thought to be influenced by the baby’s and parents’ and caretakers’ personalities, family context, economy, general environment, and cultural traditions [16]. Research emphasizes the importance of a good alliance with healthcare professionals when establishing safe and development-promoting sleep [17]. Although there is a large volume of research related to infant sleep safety, duration, and sleep hygiene, there is a lack of research exploring how health professionals can strengthen parents’ experience of self-efficacy, and how this relates to the way sleep-related advice is communicated. We find few publications from North European countries other than the United Kingdom (UK). Northern Europe is characterized by rapid demographic changes, as in most of the world [18]. Because of education or work, young people frequently move away from their families. This may weaken their social networks in the phase of life in which they become parents. Living conditions, sometimes in small apartments, may provide limited opportunities for safe infant sleep. Northern Europe is a region with a colder climate and greater seasonal variations in light and temperature than Mediterranean and tropical zones. Seasonal light shifts and polar nights are known to affect sleep among adults [19]; however, little is known about the degree to which these factors affect infant sleep development and habits. Several Northern European countries are recognized as having some of the best health welfare systems in the world [20], but we have been unable to find information that links health system quality to follow-up systems for infants and new families.

Existing safe infant sleep advice seems to be based on at least two strong paradigms [21, 22]: the risk-elimination approach advocated by the American Academy of Pediatrics (AAP) and a risk-reducing, cultural, and neurobiological approach that addresses what is thought to be natural for human beings [23, 24]. Advice and counseling must be relevant for parents and conveyed in a sensitive and understandable way, taking into consideration cultural differences [25]. There seems to be a lack of studies addressing which topics of sleep advice should be addressed and when. When screening publications over the last 20 years, we detected interventions initiated during pregnancy [26], maternity wards [27], and postnatal care [28, 29]. Research shows some inconsistencies in advice and the factors that influence parents` adherence to safe sleep guidelines, and many parents do not use safe sleep practices at home [30, 31]. Preventive communication about infant sleep includes many topics, and short interventions seem to have the best effect on parental adjustments to infants’ sleep [32, 33].

It is important to understand what healthcare professionals who meet expecting and new parents through pregnancy, childbirth, and postnatal care follow-ups should consider when providing support and information. Altogether, these services aim to promote both safe and developmentally preferable sleep in infants and healthy role transitions for mothers, partners, and families [34, 35]. These topics are addressed in this review in an attempt to provide an overview of different aspects that may be included in coherent, seamless services for new families. The topics addressed are also consistent with health-related goals launched by the Department of Health and Care, UK [36]. The research question guiding the review is as follows: “*Which factors are described as important in health personnel’s guidance of expectant and new parents when the goal is safe and well-consolidated infant sleep?*

## Method and material

The theoretical framework for scoping reviews has been known for fifteen years [37]. This methodology is recommended for investigation of complex phenomena and allows different sources of information to be selected, depending on their relevance to the questions asked. Scoping review is characterized by a processual work where all parts must be systematic, transparent, and credible. The methodology is reframed in recent years by a research team affiliated to the Joanna Briggs Institute (JBI) [38–40]. The original methodological steps focused on defining a research question, systematic searches followed by reasoned selection of studies, summarization and report of the results [37]. The updated framework emphasize that an analysis of the findings is made and that this is an essential part of further presentation of results. In line with the JBI recommendations a research protocol has been formed and published on Open Science Framework (OSF) [41]. This study deals with interactions between different groups of individuals, as displayed in the search strategy (Table 1). This study focuses on sleep concepts and factors described as important for health professionals to address in dialogues about infant sleep, both prenatally and after birth. These contexts, where parent-infant dyads meet with health practitioners, might vary somewhat depending on the health service organization in different countries. This review focuses on regular health-promoting follow-up services for new or expecting parents without known illnesses or risks. Infant sleep information and guidance may be taken care of by different groups of professionals in pregnancy (by midwives or general practitioners [GP] physicians), maternity wards (by midwives, nursery nurses, or pediatricians), or during infancy (by midwives, health nurses, GP physicians, or other services). In this paper, they are all named health practitioners, and guidance may be given as instructions, information, discussions, etc.. This review is not designed to recommend any form of guidance; thus, we address this as a communicative interaction.

**Table 1:** Data search strategy

Population	1. Fetus or newborn or infant or baby or babies
	2. Parent* or mother* or father* or pregnan*
	3. 1 or 2
Concept	4. MH sleep hygiene
	5. Infant sleep or sleep location or sleep habit or sleep hygiene
	6. 4. AND 5.
	7. 3. AND 6.
Context	8. Parent* guidance or parent* information or parent* psychoeducation or parent* support or parent* intervention or parent* involvement or parent* communication
	9. 7. AND 8.
	10. 9 AND sleep included in abstract or title

Infant sleep outcomes may be bidirectionally influenced by parental mental health and nutritional arrangements during infancy [42, 43]. The authors recognize the importance of these factors, but choose to focus solely on infant sleep outcomes. Table 2 summarizes the criteria that guided systematic searches of the PubMed, CINAHL, and PsycINFO, databases. Table 1 shows the search strategy used.

**Table 2:** Inclusion and exclusion criteria for data selection

	Inclusion criteria	Exclusion criteria
Study design	Systematic review, literature review, metasynthesis, prospective study, clinical trial, qualitative research	Professional articles, research protocol, comments, conference abstract, book, web page
Time of publication	2010 until August 2021	
Publication language	Peer-reviewed article in English, Danish, Swedish or Norwegian	Articles published in ofter languages
Place published	Indexed in PubMed, PsykINFO or CINAHL or detected in references from selected papers	
Population	Families with healthy term-born infants aged 0–6 months living together.	Prematurity, children diagnosed with diseases or specific medical problems, disabilities or institutionalized because of caregiving needs
Delimitation	The words sleep* and newborn or infant* or baby or babies were included in abstract/title	Abstract or title did not include words focusing on sleep or target population

**Fig. 1:**
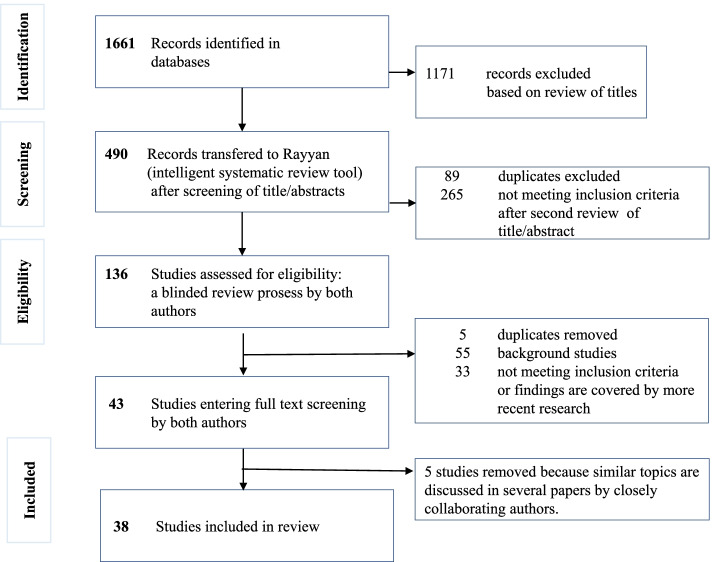
PRISMA ScR flow-chart

This review focuses on infant sleep and parental advice in countries characterized by a subarctic or continental climate, corresponding to European countries north of the Alps. However, we considered research from all the geographic regions. Additional searches have been conducted using terms as “seasonal, season, polar nights, cold climate” in combination with infant sleep. This iterative search process is in line with updated methodological advices [39]. The included databases enabled us to detect publications in the most relevant fields of research, such as nursing, midwifery, child health, medicine, and psychology. Owing to the large number of papers addressing infant sleep in this multidisciplinary field, no publications older than 2010 were considered. This limitation also seemed appropriate because previous research is largely included in later reviews. An overview of the data-selection process is presented in Fig. 1.

After importing the items to Rayyan [44], two reviewers performed blinded examinations and assessments of each source and their relevance for inclusion in repeated steps. Disagreements were resolved by discussions and repeated review of the title, abstract, and, in some cases, screening of the full-text versions. The scope of this process is illustrated in Fig. 1.

The thematic focus of the review is identified as complex, involving many environmental, parental, and child factors, in addition to relations between different participants [16, 22]. While working on the study protocol, we decided to use a framework adapted from a well-known transactional model of infant sleep to guide data extraction from each study and summarize topics and factors [16]. This model seems helpful and enables us to discuss how different aspects involved in the facilitation of infant sleep relate to each other.

## Results

Tables 3, 4, and 5 present 38 selected articles. Information about the first author, country, year of publication, aims and participants, main results, key factors, and possible implications are listed as requested [45]. More than half of the selected studies originated from the USA (21 studies), while seven were published in Oceania, eight in Europe, one in Asia, and the last was a collaborative product of European and Asian authors.

**Table 3:** Studies regarding infant sleep safety (Alphabetic ordered after first author)

	Study	Aims & participants	Methods	Results	Comments and clinical implications
1.	Baddock et al. (2019) [46] New Zealand	Investigation of infant physiology and risks and benefits of parent-infant bedsharing. The review included 40 papers reporting on infants aged six month or younger. Studies were published between 1994 and 2017.	A systematic review. Some type of objective data was assessed in 27 papers and subjective data in 32 papers.	Sleep duration was reported in 19 studies. Compared to solitary sleeping, bed-sharing was associated with significantly shorter sleep among infants in six studies, no difference in four and longer sleep duration in two studies. Bed-sharing was associated with slightly higher axillary and skin-temperature and more frequent breastfeeding. Bed-sharing was associated with lower cortisol response to a mild stressor (bath) at five weeks but not later in infancy.	*Bedsharing or not* Different sleep-arrangements influence infants sensory and physical micro-environment in different ways. Differences related to sleep architecture, infant arousals, infant overnight temperature control, cardiorespiratory responses, breastfeeding duration, sleep position, mother-infant interactions and physiological responses to mild stress.
2.	Canter et al. (2015) [32] USA	Assess the usefulness of an educational video about infant safe sleep that was tested by 43 new mothers, while 49 new mothers received standard care *N* = 92	Prospective, pre- post intervention study. Maternal observations of infant sleep were collected on surveys.	Compared to the standard care-group, video-mothers reported more frequently that they had observed appropriate infant sleep positions in the nursery (67.4% vs. 46.9%,(*p* < .05). The study did not control for changes in sleep knowledge among health workers in the unit.	*Safe infant sleep educational tools* The 6 minutes long video focused on (a) avoiding smoke, alcohol, and drugs, (b) the importance of a comfortable sleeping temperature, (c) appropriate dressing and sleep position for babies and d) the ABCs of safe sleeping: alone, on the back and in a crib.
3.	Cullen et al. (2016) [47] USA	Identify teaching strategies that nurses can use to increase families safe sleep practices. Among 100 studies, 24 papers were selected for intensively review and 14 studies included.	A systematic review and meta synthesis.	Analysis resulted in 14 findings that formed 4 themes. Meta-aggregation resulted in two synthesized outcomes: First, parents practice co-bedding with their infant despite knowledge about the risks and having received teaching. Next, families should receive co-bedding messages tailored to their specific circumstances and risks.	*Personalization of safe infant sleep guidance* For the first outcome the categories were” Keeping their babies close” and “My grandma says - family traditions”. The second outcome was obtained from two categories that covered seven findings. The categories were “Nurses influencing parenting behavior” and “Modeling ways to reduce risk”. Thus, healthcare workers should be context sensitive and personalize their information.
4	Hauck et al. (2015) [48] USA	Description of parental knowledge and practice before and after implementation of an educational program and free crib distribution. *N* = 3303 (antenatally), 1483 (postnatally) and 1729 at 3 months follow-up.	Prospective cohort study evaluating a program in a high-risk US area. Data collection by surveys developed for this study	Parental knowledge about safe infant sleep increased after receiving a crib (*p* < 0.001). Intended use of supine position for infant sleep increased from 80% (postnatally) to 87% after receiving a crib (*p* < 0.001). Unintended bedsharing decreased after participation in the program and 90% reported that the infant slept in the crib after the program compared to 51% before.	*Safe infant sleep educational tools* The authors state that programs like the BBB should be evaluated on a periodic basis to assure that they positively influence parental knowledge and practices about safe infant sleep. The program focused on sleep position, use of pacifier, bedsharing and feeding practices.
5.	Kellams et al. (2017) [49] USA	A multisite quality improvement intervention was implemented in 8 US-maternity wards, aiming to improve health professionals’ knowledge and role modeling about infant safe sleep.	A knowledge-campaign lasting for a median of 160 days (range, 101–273) across the 8 units. New mothers answered a survey before discharge from hospital	Mothers who reported receiving information on 4 primary safe sleep topics raised from 72 to 95% of the time (a 24–57% increase over the baseline). Additionally, 93% of infants were observed in a supine sleep position, and 88% of infants were observed in a safe sleep environment (a 24 and 33% increase over baselines). These rates sustained until 12 months later.	*Safe infant sleep educational tools* The intervention included information on safe sleep positions, absence of objects in sleep environment, introduction of pacifier for sleep once breastfeeding is established and room-sharing without bed-sharing. Tool kit for hospitals: PP, Posters, pocket-sized cards for nurses to use when counseling parents and sample letters to inform hospital leaders.
6.	Mathews et al. 2016) [50] USA	Can enhanced information reduce the use of soft bedding? Comparison of two groups of African American mothers receiving either standard or enhanced information *N* = 1194.	A randomized controlled study (RCT). All data are based on self-reports	Across 3 follow-ups (2–3 weeks, 2–3 months and 5–6 months) mothers in the enhanced information group used less soft bedding last night (adj OR = 0.74 (0.58–0.94), *p* = 0.013, used less soft bedding in the last week (adj OR = 0.70 (0.54–0.90), *p* = 0.006. More maternal self-efficacy was associated with more frequent inappropriate arrangements.	*Soft bedding, personalization of safe infant sleep guidance* The study focuses on a group of US-mothers that mostly were unmarried (80%) and in the lower range regarding socio-economic resources (SES). The results may be affected by high attrition rates, only 46% participated at 6 months. Mothers that left the study were younger and with lower SES.
7.	Mileva-Seitz et al. (2017) [21] The Netherlands	The prevalence of parent-child bed sharing (P-CBS) is investigated across 659 research articles (peer-reviewed, editorial pieces and comments).	A systematic review.	P-CBS was reported in 98 studies. Prevalence ranged from 2.5% in an Australian study to 100% in many African countries. The relation between P-CBS and risks of sudden infant death is investigated and nuanced. The reviewers call for collaboration across different disciplines introducing a new term “psychoanthropediatrics”.	*Intentional or reactive bedsharing* Some studies focus on the difference between intentional and reactive bed-sharing. Results indicate that intentional bed-sharing parents were more likely to bed-share all night, to endorse and be more satisfied with bed-sharing, while parents of reactive bed-sharers had more often tried a ‘cry-it-out-method’ of sleep training and reported more night-time difficulties.
8.	Patton et al. (2015) [30] USA	Investigation of whether nurses provide safe sleep environments for infants in a hospital setting Sample sizes ranged from 94 to 5911 nurses and/or other healthcare workers across included studies. Samples of parents ranged from 100 to 671.	An integrative review based on search in multiple databases (1999–2013) 16 studies included.	Four papers discussed nursing knowledge and compliance with safe sleep recommendations. In 12 of the 16 studies incorrect recommendations from nurses were detected. Most nurses are aware of the AAP recommendations but may choose not to follow them consequently due to fear of aspiration. Parental observations mirror these inconsistencies in health workers’ practice: 50% used the side-lying position, 37% the supine position, and 10% a combination of positions.	*Safe infant sleep education and role modeling* The choices nurses make regarding safe infant sleep practices can influence parental behavior at home. In the included studies some mothers were not even aware of sleep related risk factors. When nurses lack knowledge and their practice is inconsistent, parents may find themselves confused. If parents observe inconsistencies between nurses’ practice and teaching, they may use the positions they observed professionals performed.
9.	Raines (2018) [31] USA	Investigates which factors that influence on parental behaviors related to newborn safe sleep positions and environments in the home.	Descriptive qualitative study. - qualitative interview conducted by telephone	Participants described three consistent factors that influenced how newborn sleep at home. The most frequently mentioned influence was *Other People* followed by *Nobody/No One* and *Images* from sources such as books, pictures, television, and the Internet.	*Safe infant sleep and parental believes* Parents need education about the rationale for the AAP safe sleep guidelines and nurses are important in helping them understand AAP recommendations for safe sleep. The images to which parents are exposed send mixed messages. Health care professionals should portray safe sleep for infants in media, marketing materials, and other graphic representations.
10.	Salm-Ward et al. (2018) [51] USA	A comparison of parental knowledge and use of safe sleep practices after distribution of an educational program and baby cribs. Mothers that either were pregnant in the last trimester or mother of a child less than 3 months old were eligible, *N* = 132.	A prospective, matched pre- and post-test cohort study with follow-up. Surveys were answered by use of telephone interviews ten weeks after post-test survey.	Participants reported increased knowledge after the program: recommended back position (58.8% at pre-test vs. 96.2% at post-test, *p* < 0.001); recommended separate surface in parents’ room (53.4 vs. 74.8%, *p* < 0.001); no soft items (85.9% vs. 96.9%, *p* = 0.001); smoke exposure increases risk (57.1 vs. 93.7%, *p* < 0.001); breastfeeding reduces risk (55.8 vs. 82.2%, *p* < 0.001); back sleep does not increase risk of choking (48.8 vs. 85.6%, *p* < 0.001); pacifier use decreases risk (9.6 vs. 62.4%, *p* < .001); always back sleep (76.4% vs. 92.9%, *p* < 0.001); and infants should sleep on a flat, firm surface (89.1 vs. 98.4%, *p* = 0.003).	*Safe infant sleep education* At pre-test, 58.4% of participants reported receiving a health workers advice on sleep position and 40.2% on sleep location. The only informational item that were significantly less known the responders at follow-up compared to the post-test survey was that use of a pacifier decreases the risk of SUID.
11.	Vilvens et al. (2020) [52] USA	Focus on why parents/ caregivers might fail to practice safe infant sleep arrangements. (*N* = 124) Parents of infants less than one year were interviewed.	A descriptive, qualitative study based on interviews. Use of ‘pulse interviewing’ -talking with parents at community events in a high-risk US area.	Six themes of underlying reasons why caregivers might not practice safe sleep behaviors were identified and included: (a) culture and family tradition; (b) knowledge about safe sleep practices; (c) resource access; (d) stressed out parents; (e) lack of support; and (f) fear for safety of baby.	*Safe infant sleep and parental resources* Based on the study information the authors formed five narratives (personas). These learning examples are introduced as resources that may strengthen health workers ability to be context sensitive and personalize their information in communication with new parents about infant sleep safety.

**Table 4:** Development and evaluation of sleep related interventions. (Presented in publication year order)

	Study	Aims & participants	Methods	Main results	Comments and clinical implications
12.	Douglas et al. (2013) [53] Australia	Investigates if behavioral sleep interventions in families with infants less than 6 months may improve child and maternal outcomes.	Systematic review. 638 studies were detected, and 43 studies selected for review.	The authors argue that behavioral sleep interventions are inefficient for infants younger than 6 months. Main critiques are presented as 3 methodical limitations related to:1) lack of focus on associations with feeding difficulties, 2) to little differentiation between the first and second part of infants first year and 3) that some studies interpret associations as causal relations.	Focus on the high variability in neurodevelopmental stages and sleep maturation the first six months. Unsettled infant behavior in this period is a complex phenomenon. They argue for a holistic approach in attempts to support and empower parents including individualized approaches, cue-based care, and healthy daytime biopsychological rhythms.
13.	Kempler et al. (2016) [54] Australia	May a psychosocial sleep-focused interventions improve infant sleep or maternal mood in the first year?	Systematic review and meta-analysis of RCT’s. 7 of 9 included studies reports on children less than six months (*N* = 2023 mother-infant dyads).	The designed interventions had a small positive effect on total night-time sleep for infants (TNTS), *p* < 0.001, Hedge’s g = 0.204, but only a weak, non-significant effect on infant night waking.	It is a great heterogeneity between the programs included in this study. Thus, they can’t conclude what types of interventions that is best. Efficacy depends on infants’ maturation and age. Results from this study differs compared to Douglas et al. (2013) [53] but are in line with previous Cochrane review [57].
14.	Cricton & Symon (2016) [55] Australia	Exam the effects of behavioral sleep techniques on infant aged six month or less.	Non-systematic review. 11 studies, covering 2663 infants included	The studies provide evidence that active preventive intervention improves infant sleep in the earliest month of life (no statistics). No results indicating harmful effects of delayed responses were found.	Key information given to parents included: infant sleep physiology, patterns and cycles, sleep onset associations, routines, use of diaries: focal feed, self-settling, settling techniques, minimized interactions on night, differentiate night and day. Provision of parental education differed between individual and group settings.
15.	Paul et al. (2016) [56] USA	Investigates if a responsive parenting intervention (RPI) might improve sleep outcomes in a sample of families with newborn infants compared to a control group. *N* = 279 infants.	RCT that recruited families right after childbirth and conducted follow-up assessments at 2, 8, 52 weeks after birth with parental reports.	Compared with controls the RPI- group were less likely to have prolonged bedtime routines at one year of age, earlier bedtimes at 16 weeks, they were more seldom fed to sleep and had longer nocturnal sleep duration at 8, 16 and 40 weeks.	RPI is a multicomponent program including advice about bedtime routines, sleep location and parental soothing behavior and behavior in relation to night waking.
16.	Mihelic et al. (2017) [29] Australia	Evaluation of the efficacy of parental interventions. Only results on infants sleeping problems are included here (13 studies).	A meta-analytic review of publications from 5 databases, all published before February 2016.	Participation in the intervention groups were associated with significantly better sleep outcomes among children, Cohen’s d = 0.24, *p* < 0.001 compared to control groups. Interventions assessing infant crying did not show significant intervention effects.	The findings concur with Cochrane review in 2013 [57]. Parental competence or confidence was only assessed in five studies and the authors mention this as these factors are known to have impact on sleep outcomes.
17.	Galland et al. (2017) [58] New Zealand	Evaluates the preventive effects of a sleep intervention given antenatally and three weeks post-partum. *N* = 802 families	RCT with four groups: 1) usual care, 2) sleep intervention, 3) activity and feeding intervention and 4) combination of 2) and 3). Data based on parental self-report and actigraphy.	Analysis with linear mixed models did not detect any significant benefits of the sleep education at 6 months. The frequency of problematic sleep reported by all mothers were 16.1% at 4 months and 19.9% at 6 months. Parental practices related to infant safe sleep were not affected by the intervention.	The authors questions if antenatal education to parents may be to early. They acknowledge that infant awaking and disrupted sleep the first 6 month should be viewed as normal, due to the ongoing neurodevelopmental maturation and infants’ adaptation to the extrauterine world.
18.	Middlemiss et al. (2017) [59] USA	Investigating changes in infants total sleep time (TST) in a prospective, clinical study. Families were enrolled in a sleep intervention program. *N* = 34 mother-infant dyads.	A response-based sleep intervention with four timepoints when children were from 4 to 11 months old. Data was collected from sleep logs, questionnaires and salvia-samples.	Analyses of infant sleep, from before admission to the program until finish detected a significant increase in childrens total sleep time (TST) (t(59) = 8.96, *p* < 0.001). The mean increase in TST was 5.8-hour pr 24 hours.	Teaching parents to understand and respond to infant cues, through the day and at the transition to sleep was associated with an increase in mothers’ ability to overview their infants sleep schedules. These effects were possible without engaging in behavioral extinction strategies. Interestingly, maternal registrations of infant sleep were associated with higher degrees of maternal anxiety or depression, possibly associated with changes in response patterns.
19.	Martins et al. (2018) [27] Portugal	An evaluation of a postnatal education program distributed to 159 new mothers (IG) before discharge from the maternity ward in comparison with a control group (CG). Total *N* = 314.	An experimental, longitudinal study with implementation of a 15- minutes educational session. Data was collected from self-report on questionnaires at childrens age of 1,2,4 and six months.	Infants in the IG group slept more often in their own bed at their age of one month (OR adj = 4.51; 95% CI,1.69–12) and were more often able to fall asleep alone (OR adj = 4.11; 95% CI, 2.3–7.4). IG-infants were less often feed to sleep than controls (OR adj = 1.49; 95% CI, 0.9–2.4). At 6 months IG-infants still were reported as more able to fall in sleep alone and needed less feeding to sleep.	The intervention consisted of one individual session with a specially trained sleep-consultant (pediatrician), supplemented with a leaflet. The information included knowledge about normal sleep cycles, the importance of sleep for healthy development, sleep hygiene including routines, infant solitary sleeping, sleep promotive environments and how to support infants self-soothing capacities.
20.	Ball et al. (2020) [28] United Kingdom	A description of the development of a new approach to support parental wellbeing and responsive infant caregiving in UK. Responses were given by 164 health practitioners and 535 new parents.	An action research study that adapts an existing Australian program to a new UK-intervention “Sleep, Baby & You” (SBY). Data were collected on stakeholder meetings. Field-testing of the SBY were done among health practitioners and new parents.	Practitioners were positive about the concepts and suggestions contained within the SBY-materiel. SBY is described as a non-prescriptive approach that search to normalize the life with a newborn infant and the challenges it brings. 12 parents gave anonymous response on the program. They emphasize the importance of getting knowledge about normal sleep development and that spoken and written materiel support each other.	Feedback given across this developmental process confirms that parents want knowledge about normal infant sleep both antenatally and in infancy, adapted to the needs of each family. This paper does not mention the importance of infant safe sleep; thus, we don’t know if it’s incorporated in the program.
21.	Sweeney et al. (2020) [26] New Zealand	A pilot testing of a perinatally delivered behavioral-educational sleep intervention (PIPIS). 20 mothers formed a control-group (CG) and 20 a sleep intervention-group (SIG). *N* = 40.	A pilot of a controlled trial. Data collection used self-reported sleep quality and actigraphic measures from mothers and infants.	Total sleep time per 24 hours (TST) among mothers were similar in the groups, but total nocturnal TST became significantly different. SIG-mothers had an increase of 47 minutes in nocturnal sleep, but not for CG-mothers from 6 to 12 weeks postpartum [t(36.55) = − 4.30, *P* < 0.001. Infants sleep outcomes were similar across groups. By 12 weeks, compared with 6-week reports, maternal perception of infant sleep problems was improved for SIG-mothers but not for CG-mothers.	SIG-mothers reported greater confidence in managing their infants sleep even the relation was not tested. Infant sleep development follows individual tracks the first six month of life. Results from this study may indicate that the intervention strengthened mothers’ capacity to handle these challenges.

**Table 5:** Studies exploring aspects associated with infant sleep quality (Alphabetic ordered after first author)

		Aims & partisipants	Methods	Main results	Key words and comments
22.	Adams et al. (2020) [60] USA	Investigation of how early or later bedtimes may influence night-time sleep duration in 2–24 weeks old infants. *N* = 24 newborns	Study with microburst longitudinal design, assessing infant sleep with actigraphy and maternal reports at infants ages 6, 15 and 24 weeks.	For every 1 hour earlier in infants’ usual sleep night-time onset (SNTO) total night-time sleep (TNTS) was 34.4 min longer that night (*p* < 0.01). Infants with earlier than usual sleep onset had slightly earlier sleep offset the next morning (8.4 min for every 1 h earlier in onset; *p* = 0.02).	*Bedtime routines; sleep duration.* This study demonstrate that earlier bedtimes may benefit sleep among the youngest infants in addition to early consistent bedtime routines.
23.	Bennett et al. (2013) [61] United Kingdom	Review of how massage may promote sleep habits among healthy term-born infants aging 6 month or less.	Cochrane review of studies published before June 2011. Inclusion of 34 RCTs.	Heterogeneity between included studies were substantial and massage was only modestly associated with positive effects on 24-hour sleep duration.	*Parent behavior (babymassage); sleep quality* No studies documented negative effects of massage for this population
24	Bilgin & Wolke (2020) [62] United Kingdom	Observation of associations between parental use of “cry it out” and childrens attachment quality and crying behavior at 18 months. *N* = 178 parent-infant dyads.	Prospective, longitudinal study recruiting families across 3 maternity wards. Parents reported use of “cry it out” at term and when infants were 3, 6 and 18 months.	No adverse impacts of leaving infants to “cry it out” was detected in the first 6 months of life. Tested in relation to infants’ attachment quality and cry-frequency at 18 months. Often use of “cry it out” was reported by 8–13% at term-age, 3 and 6 months. Never use of this strategy were reported by 63% at term-age and from about 40% of families at 3 and 6 months.	*Parent behavior and infant crying.* Leaving infants to “cry it out” at term, both a few times and often, was negatively associated with frequency of crying at 3 months. The authors neither recommend leaving infant to cry out nor responding immediately. The findings are thought to be consistent with an approach to parenting that is intuitive and adapts to infants demands and regulatory behaviors.
25.	Bruni et al. (2014) [63] Italy	A longitudinal examination of sleep patterns, habits and parent-reported sleep problems among infant aging 1 to 12 months. *N* = 704 infants assessed by 81 different Italian pediatricians.	Prospective, longitudinal cohort study collecting data on sleep outcomes from parents answering surveys by telephone interviews. 3520 surveys were collected.	Both night-time, daytime and total sleep duration showed high inter-individual variability from one to 12 months associated with changes the first six months and stability after that	*Sleep quality; sleep duration* Investigates sleep arrangements and sleep challenges. Provides a context for clinicians to discuss sleep issues with parents and suggest that prevention efforts should focus to the first 3–6 months, since sleep patterns show stability from that time point to 12 months.
26.	Chen et al. (2019) [64] Singapore & Germany	An investigation of associations between screen viewing (SV) and sleep duration among children less than 2 years old. *N* = 714 infants.	Cross-sectional study using parental reports of SV and sleep.	For the whole sample was 1 hour SV associated with 16 minutes shorter TST (*p* < 0.001). The prevalence of daily SV among infants 0 to 6 month was 28.3% and their mean screen time was 60 minutes (IQ range 0.44 to 120) Compared to the no-screen group a dose-response relation was detected for young screen-viewers.	*Screen viewing; sleep duration* The negative association between SV and sleep duration was strongest among the youngest infants.
27.	Field et al. (2016) [65] USA	An investigation of differences between baby massage with lotion (LM-group) or without lotion (non-LM group) or no massage at all (control group) and whether this influences infant sleep quality. *N* = 76 mother-infant dyads.	RCT, program started in the post-delivery ward and was evaluated at a 1-month follow-up. Use of maternal self-report.	Compared to the other groups, shorter sleep latency was found in the LM-group (*p* = 0.002), and decreased number of nightwakings (*p* = 0.008). LM-mothers reported higher confidence in managing their infants sleep (*p* = 0.03). There was a positive correlation between the number of massages and longer infant night-time sleep (*p* < 0.005).	*Parent behavior (babymassage)* Infant and maternal sleep was highly correlated. Massage without lotion might be uncomfortable for newborns and new parents need to get knowledge about this factor and the dose-dependent association between baby massage and night-time sleep. Maternal ratings of infant sleep difficulties tended to decrease in the LM-group compared to the non-LM group.
28.	Fiese et al. (2021) [14] USA	An investigation of associations between bedtime routines and infant sleep quality in infancy. N = Parent of 468 infants.	Part of a large prospective, longitudinal cohort study. Bedtime routines and sleep measures were reported by mothers on questionnaires at infants ages of 3,12,18 and 24 months.	More bedtime routine consistency predicted less night-time waking and sleep problems across infancy. More adaptive activities at 3 months of age predicted longer sleep duration at 12 months (r = 0.12, *p* < 0.05).	*Bedtime routines; sleep quality* Bedtime routines and sleep outcomes had moderate stability over time, especially from one to two years.
29.	Kahn et al. (2021) [66] USA	An examination of the moderating role of infant age on the relation between infants’ use of different screens and sleep outcomes. *N* = 1074 infants aged 0–18 months.	Cross-sectional study. Data collection by parental reports and autovideosomnography.	Age moderated the relation between daytime touchscreen exposure and sleep. For 3- months old infants were a 5-minute daytime touchscreen exposure associated with an average decrease of 13 minutes daytime sleep (b = − 2.62, SE = 0.62, *p* < 0.001. More daytime touchscreen exposure was associated with fewer awaking’s (b = − 0.03, SE = 0.01, *p* = 0.03)	*Screen viewing; sleep duration* The author suggests that displacement of daytime sleep by screens may lead for a greater accumulation of sleep homeostatic pressure, possibly facilitating more consolidated night-time sleep among the youngest infants.
30.	Lennartsson et al. (2016) [67] Sweden	An evaluation of a child health nurses educational program aiming to improve their ability to talk with new parents about risks of cranial asymmetry in newborns and how to prevent this the first 4 month after birth. *N* = 272 parents	A cross-sectional survey nested in a larger intervention study. Data collection by parental report on questionnaires at children’s age of 4 month.	Parent in the education-group reported more often that they were aware of the importance of alternating the direction of their infant’s head when initiating sleeping compared to control-group parents (82% versus 64%, *p* = 0.001). Significant group differences were also found in relation to which kind of pillows that could be used and when they should be removed.	*Parent behavior; Infant physical health* The paper gives detailed information about the advice nurses should give and it seems relevant to integrate this in talks about infant sleep issues.
31.	Meyer et al. (2011) [68] Germany	A comparison of sleep among healthy infants. A swaddling group (SG = 40) and a control group using sleeping bags (CG = 45) were compared. *N* = 85 infants.	Prospective, observational study with use of polysomnography to assess differences in sleep stages and awaking’s in 7.5 weeks old infants.	Compared to use of sleeping bags swaddling reduced the rate of spontaneous waking (*p* = 0.020), reduced the number of changes in sleep state (*p* = 0.015), promoted more quiet sleep (*p* = 0.032), and reduced time spent awake (p = 0.001). Sleep efficiency was increased (p = 0.001).	*Parent behavior (swaddling); sleep quality* The authors conclude that swaddling may reduce the risk of infant injuries or death as sleep becomes more consolidated even when children sleep in the recommended supine position.
32.	Mindell et al. (2015) [69] USA	Investigates if there are dose-dependent associations between use of bedtime routines and healthy sleep habits. N = 10,085 mothers from 14 countries.	Multinational cross-sectional study using the Brief Infant /Child Sleep Questionnaire (BICQ) in all countries.	Consistent bedtime routines (CBR = 3–7 times per week) among preschoolers were associated with more routines in infancy. A dose-dependent association between CBR and better sleep outcomes were documented for infants, toddlers and preschoolers.	*Bedtime routines* Routines that are instituted in early infancy are associated with better sleep outcomes later in childhood.
33.	Mindell et al. (2018) [70] USA	A study examining the impact of a consistent bedtime routine involving massage on infants and mothers sleep and mood. *N* = 123	Prospective study randomizing families with 3–18 old infants to an intervention (IG, *n* = 64) or a control group (CG, *n* = 59). Data collected by maternal self-report.	After one and two weeks no changes were detected among CG-infants while night-waking decreased among IG-infants at both times (F = 5.36, *p* = 0.006). Fewer IG-mother reported their infant with a sleep problem at these times (*p* = 0.013) and they were more confident with their ability to manage infant sleep compared to CG-mothers (F = 8.42, *p* < 0.001).	*Bedtime routines* Participating families were not identified with any sleep problems before study inclusion. Mean age of the children were almost 9 months. The intervention did not affect any other maternal outcome than fewer night-waking.
34	Oden et al. (2012) [71] USA	Investigates the associations between parent’s use of swaddling and infant sleep positions. *N* = 103 parents	Cross-sectional descriptive study recruiting parents to answer a survey at well child visits with infants aged 0–3 months.	All parents had sometimes swaddled their infant and parents who used swaddling routinely were more likely to but their infant in supine position when swaddled (*p* < 0.01).	*Parent behavior (swaddling)* Most parents found swaddling as a safe practice. Parents who not swaddled routinely were more likely to use inappropriate sleeping positions. Parents need to learn how to swaddle in a safe manner to avoid increased risks of injuries or sudden death.
35.	Paul et al. (2017) [72] USA	This study investigates associations between parent-infant room sharing and sleep outcomes. *N* = 230 mother/infant dyads with healthy, term born infants at a US maternity ward.	A randomized controlled trial collecting data by maternal self-report. Mothers reported on Brief Infant Sleep Questionnaire, BISQ) at children’s age of 4 and 8 months	By 4 months: children sleeping in separate rooms had more regular bedtime routines adjusted OR 1.93 (95% CI:1.05–3.53,), p = 0.03, they were put to bed earlier adjusted OR 1.93 (95% CI: 1.06–3.53), *p* = 0.03. They were also reported with longer sleep laps (29 min.) than room sharers (p = 0.02), fewer feedings at night (p = 0.02), safer sleep environment (p = 0.02) and less frequent moved over to parents’ bed at night adjusted OR = 0.24 (95% CI: 0.09–0.61), *p* = 0.003	*Sleep location; sleep quality* This research is one part of a large comprehensive study that primarily focuses on obesity prevention.
36.	Philbrook & Teti (2016) [73] USA	Investigates associations between night-time parenting behavior and infants’ cortisol patterns at 3,6 and 9 months. *N* = 82 mother-infant dyads.	Prospective, observational study collecting data by use of video equipment in family homes, salvia sampling and maternal self-report.	Multilevel modeling revealed that infant cortisol levels were lower at timepoints when mothers were scored as emotionally available (b = −.25, *p* < 0.01). This association was driven by lower infant cortisol levels at bedtime (b = −.35, *p* < 0.01).	*Bedtime routines; parental emotional availability* Findings shed light on factors involved in transactional relationships between infants and parents, relevant in relation to stress regulation and the importance of biobehavioral synchrony. Cortisol decreased with age for infants of less emotional available mothers but remained at low levels for infants of more emotionally available mothers.
37.	Voltaire & Teti (2018) [74] USA	An investigation of associations between different sleep arrangements (cosleepers or not) and two different night-time behaviors among parents (using distress-initiated or non-distress-initiated strategies). *N* = 107 families	Prospective observational study (part of the large SIESTA-study). Families were recruited in the maternity ward and data collected at children’s age of 1,3,6 and 9 month using observations and questionnaires	The number of distress-initiated parent-infant episodes at night in the first 3 months affected the decline in nightwakings. More of these episodes were associated with a steeper decline in night waking. For infants that mostly were solitary sleepers more non-distressed episodes were associated with slower decrease in number of night-waking with age and low levels of non-distressed episodes were associated with faster reduction of night waking.	*Parenting behavior; physical health; sleep quality* It is suggested that infant sleep regulation may benefit from appropriate night-time interventions in the 3 first month of life, while similar association not were detected at six months. Maternal responsiveness during the first few months of life is associated with lower levels of infant distress toward the end of the first year.
38.	Östürk & Temel (2019) [75] Turkey	A 90-minute training program focusing on appropriate soothing techniques for newborns were introduced to an intervention group (IG = 21) at a home visit 4 weeks after given birth and compared with families forming a control group (CG = 21). *N* = 42 mother-infant dyads.	A single-blind randomized experimental study comparing IG and CG parents reports of infant’s self-regulation when infants were 3,7,11 and 23 weeks old.	No group differences were found before the program was introduced to the IG group. At 7 weeks IG-infants slept more than CG-infant (on average 78 min per 24 h, *p* = 0.004), woke up more seldom (*p* = 0.001) and cried less (p = 0.001) and fed more seldom per 24 h (p = 0.001). Similar group differences were detected at infants age of 11 weeks, and for most variables, but not for sleep duration at 23 weeks.	*Parent behavior (soothing techniques); sleep quality* Helping new parents to understand how they can support infants to establish best possible regulative behavior across the first weeks and month of life seem. The techniques used in this study were swaddling, holding an unsettled infant in side- or prone position, use sushing with white noise and swing the infant vertically.

**Fig. 2:**
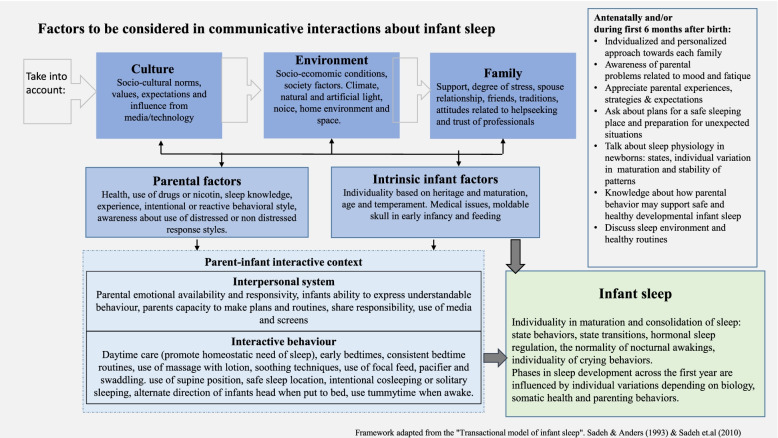
Factors to be considered in health practitioners’ communicative interactions with new or expectant parents

The included studies shaped three main categories of information that answered our research question. Table 3 presents 11 studies on infant sleep safety (7 original studies and 4 reviews). Table 4 presents nine studies describing complex or specific infant sleep interventions, relevant until infants age of 6 months (five original studies and four reviews) and Table 5 presents 17 studies exploring factors that may have an impact on infant sleep quality and duration until 6 months of age (16 original studies and 1 review). Quality assessment of the included studies is not obligatory when conducting a scoping review. However, the detection of recently published high-quality papers has become a priority in the selection process because several important sleep-related factors have been investigated in many original papers and reviews over the last 10 years. The factors suggested for incorporation into health practitioners’ communicative interactions with new parents are listed in Fig. 2. Unfortunately, additional searches focusing on sleep and climatic factors, such as polar nights and seasonal lightning, did not identify studies that qualified for inclusion in this review.

## Discussion

This study investigated which factors are described as important in health practitioners’ communicative interactions with new and expectant parents on infants’ sleep safety and development. We recognize that topics related to parents’ health and their decisions regarding infant feeding are interwoven in the planning and nurturing of infant sleep. However, these factors are not discussed in this review. The following discussion is based on the three categories described in Tables 3, 4 and 5 and refers to many of the factors summarized in Fig. 2.

### Studies regarding infant sleep safety

Table 3 includes 11 studies that investigated the effects of interventions promoting safe infant sleep in different educational programs. Seven studies described interventions based on the AAP guidelines which highlight the importance of back to sleep for infants, use of pacifiers in the first year, avoidance of soft sleeping surfaces, and items in infant beds that can increase the risk of SUID and warnings against parent-child bedsharing [24, 50]. Both short interventions, such as educational videos, and longer programs and campaigns have been described [32, 49, 51]. Four studies have discussed earlier research findings on similar topics.

Parents’ capacity to receive information in the perinatal period is described as limited [47, 48]; thus, how information about and modeling safe sleep issues are personalized and given is important [52, 76]. Health practitioners’ teaching strategies must be tailored to fit each individual family for success [47]. Several studies have emphasized the importance of updated knowledge and consistent practices among health practitioners [30, 49]. One important finding is that health practitioners in maternity wards should be educated in safe sleep practices and perform according to what they advise parents to do [30, 49]. By providing safe sleep practices in a hospital setting, health practitioners can influence parental behavior through modelling with their own practice [30, 31, 47].

Although highlighted safe sleep recommendations seem to be widely known and recommended, research shows that parents tend to form their own practice in their home environments [22, 28, 29, 47]. This applies particularly to the sharing of the same bed surface. Parents are influenced by family, friends, and society [16], as well as advertising and the Internet [77], and they frequently practice bedsharing despite AAP-recommendations not to do so [24]. In Europe, recommendations regarding parent-infant bedsharing (P-CBS) are articulated somewhat differently compared to those in the USA, depending on national policies [77–79]. Bedsharing is neither recommended nor discouraged in the UK. Swedish recommendations state that infants younger than three months should sleep in a cot, while Norwegian recommendations focus on how parent-child bedsharing can be performed safely [77–79]. Recent research has reported that bedsharing is not associated with an increased occurrence of SUID when other risk factors are considered [80]. Recent Scandinavian studies have reported increasing rates of bedsharing [4, 81]. A risk-reducing approach may thus be more acceptable in many European countries, as health practitioners recognize that co-sleeping occurs, and their advice can focus on how to facilitate intentional bed-sharing securely. This seems important, as reactive or spontaneous unplanned bed sharing is associated with an increased risk of injuries or SUID [21]. It seems important to share knowledge with parents in a non-judgmental way regarding how bedsharing may affect infants’ physiology. Cot versus co-sleeping arrangements influence sleep outcomes, such as sleep architecture, arousal, and overnight temperature control [46]. Longer sleep durations have been reported among solitary-sleeping infants than among co-sleepers [80]. The last finding may be biased by the fact that co-sleeping mothers seem to recognize night waking among their infants more easily than non-co-sleeping mothers [23].

Many families live in space-restricted apartments. This may limit the use of a separate baby bed. A cot-distribution program that included early antenatal communication reported positive outcomes [51]. In a study investigating the use of cardboard boxes distributed through a pediatric primary care clinic in the United States, more than half of new parents reported that they would use the cardboard box for their infant to sleep in if provided [82]. Considering the family environment (Fig. 2), the distribution of cardboard boxes can also encourage safe sleeping in families with low economy [82]. Finland has long traditions in supplying new families with a card board box to encourage safe sleep, and is known to have low SUID rates [83].

The purpose of parental guidance is to empower parents and address their own expectations, experiences, and opinions as well as family culture while sharing sleep-related knowledge with them (Fig. 2) [16, 22]. One communication topic may be that the use of a pacifier while sleeping not only affects the infant’s regulative behavior, but also reduces the risk of SUID, possibly because repeated sucking promotes more light sleep [31, 48, 49, 84]. When parents are offered understandable and knowledge-based advice, it may be easier for health practitioners to establish trustworthy relationships with new families. When respectful alliances are formed, parents may be more likely to find advice and recommendations relevant to them [35]. Our review shows that guidance and recommendations can be promoted through different approaches, which should be consistent and knowledge-based.

### Development and evaluation of interventions addressing infant sleep consolidation

Six studies, published in the last 5 years, describe infant sleep-related interventions with high relevance for the early postnatal period [26–28, 56, 58, 59]. In addition, four reviews summarize earlier studies and discuss the degree to which sleep-related interventions may increase sleep outcomes in the postnatal period of life [29, 53–55]. One intervention was started antenatally, two were implemented in maternity wards, and three were designed to support families at postnatal follow-up meetings.

Different theoretical frameworks appear to inform the design of the interventions and studies included. Several interventions aim to teach parents about infant sleep and how they can promote early consolidation of sleep habits in their infants [27, 56, 58, 59]. Ball et al. (2020) introduced an alternative approach focusing on parents’ need for knowledge about normal sleep development, homeostatic sleep pressure, healthy activities when awake, the importance of cued-based care, and that advice should be adapted to each family and their cultural preferences [28].

This review will not explore the gaps between different approaches, but some gaps seem important and may reflect the different conclusions reported in the reviews included [29, 53, 55]. A review from 2013 concluded that behavioral sleep interventions during the first six months of life do not have significant effects on infant sleep [53], while later reviews reported several positive effects [29, 54, 55]. Some studies found that early interventions may increase infants’ night-time sleep [26, 54], reduce the frequency of nightwakings [71], increase the use of recommended early bedtimes for infants [56], prevent prolonged bedtime routines, eat as the last activity before sleep [27, 56], and help infants to sleep more regularly in their own cot [27]. No intervention reported effects related to the duration or frequency of infant crying. This concurs with a previous suggestion that infant crying in the first months of life depends on the maturation and biology of each individual child [57].

### Studies investigating aspects associated with infant sleep quality

The final group of studies consisted of 16 original studies and one review addressing different sleep-related factors (Table 5). All studies focused on aspects of the parent-infant interactive context related to sleep quality (consolidation) or infant sleep duration (Fig. 2). Parent behavior related to bedtime routines and/or night-time behavior has been addressed in six studies [14, 60, 69, 70, 73, 74]; two studies focused on the benefits of regular babymassage [61, 65] and two on the benefits of regular swaddling [68, 71]. One US study reported results related to parent-infant sleep and room sharing [72], one Turkish study reported significant improvements in infant sleep after teaching parents different soothing techniques [75], and two studies reported somewhat contrasting findings related to associations between infants’ daytime screen-viewing and infant sleep [64, 66]. One study report findings related to the impact of parents’ emotional availability in interactions with their child prior to sleep [69], while another report that parents use of “cry it out” strategies is not associated with negative outcomes at children’s age of 18 months, even when this strategy has been used across the first month of life [62]. One study addressed the importance of varying the direction of the infant’s head when initiating sleep because the skull of a newborn baby is soft, thus increasing the risk of cranial asymmetry [67]. Parents need appropriate information about this, and an education program designed for nurses has been reported with promising results [67]. Advice on the use of the correct type of pillow may conflict with safe sleep recommendations. Lastly, an Italian study confirmed previous findings of much more variation in infants’ sleep consolidation in the first versus second half of the first year [63].

The studies mentioned above were selected because they tap different aspects highlighted in the transactional model of infant sleep [8, 16]. Thus, it is important to consider when health practitioners design communicative interaction programs for new parents. Many studies confirm the importance of consistent bedtime routines as early as possible during the postnatal period [14, 60, 65, 69, 70, 73]. The importance of consistent, multicomponent routines flexibly adapted to each child may not be obvious to new parents. Parents may need information about the advantage of putting a newborn to sleep drowsy but awake, or to be introduced to the use of babymassage with lotion as a part of a routine, along with other elements fitting in with their traditions and values [47, 52]. Many parents need basic information about massage rather than touch and tender patting of the skin [15, 65, 69, 70]. The need for more knowledge may also apply to swaddling [68, 71].

The factors mentioned above may influence both safe sleep arrangements for infants and how parents can support the optimal development of regulative behavior and sleep consolidation in their child [31, 47, 71]. Thus, interventions for new or expectant parents and infants should combine communication about safe infant sleep and advice about how they can influence sleep consolidation in their child. This new paradigm was introduced by Mileva-Seitz and colleagues [21]. The holistic and cue-based intervention SBY, designed in the UK in close collaboration with many stakeholders, seems to be a good starting point [28]. Nevertheless, we could not identify any focus on infant safe sleep issues in the SBY program description. A recurrent theme across included studies is the importance of giving parents knowledge about newborn infants’ sleep behaviors and states, and that these patterns differ significantly from sleep in older infants and adults [26, 55, 58, 75]. A similar focus is prominent in the framework of newborn behavioral observation (NBO) [2, 35], where health practitioners are educated on how to help parents understand and adapt to their children’s sleep and other behaviors. NBO focuses on how to understand infant behaviors as meaningful expressions, helping parents adapt to them, and finding helpful parenting solutions. The NBO approach appears to be a powerful tool to consider when designing new sleep-related interventions. Even in the first months of life, when rhythms involving eating, sleeping, and social interactions are characterized by a lack of regularity, parents seem empowered when they discover their infants’ incipient competences [85].

Another recurrent theme is the importance of individualized and personalized guidance by parents on infant sleep issues, avoiding standard information packages [47, 52]. This is in line with a Scottish qualitative study of how new mothers want to be met and supported by health practitioners [86]. Mothers want to work in partnerships with professionals, obtain knowledge-based advice free of stigma, help establish realistic expectations, and encourage them to make their own choices based on their family’s needs [86]. This is almost a rewriting of the values promoted by NBO courses in Norway [35]. The famous words of the Danish philosopher Soren Kierkegaard, referred to as “the art of helping”, may be the best way to sum up this important theme: *“If one is truly to succeed in leading a person to a specific place, one must first and foremost take care to find him where he is and begin there. This is the secret in the entire art of helping”* [87].

One important reason why infant sleep becomes a challenge for new parents is short naps, night waking, and frequent infant crying behavior [88]. Several of the included studies dealt with crying behavior. High frequencies of infant crying have been reported in studies in many different countries and cultures, even though the amount of crying has been reported to be significantly different between nations [89]. Crying behavior is closely related to conditions in an individual infant, and systematic reviews have failed to detect interventions that significantly reduce crying behavior [57]. While some describe strategies such as delayed response or letting the baby “cry it out” as possibly harmful [28] one study reports no long-term negative effects of “cry it out” in the first six months of life on childrens’ attachment quality [62]. Philbrook and Teti reported that parents’ emotional availability may be more important than how they respond when settling an infant to sleep [73]. Another study reported that appropriate nighttime interventions in the first three months of life may support infant sleep consolidation. Non-distress-initiated interventions seem to disturb solitary sleepers differently compared to co-sleeping infants [74]. Different findings do not shape a clear picture, but several studies show that swaddling and soothing behaviors may reduce infant crying and fussiness [71, 75]. More research is needed to investigate whether or how different aspects of parental behavior influence infant sleep across the four quarters of the first year of life. A recent Finnish study documented that natural and artificial light have an impact on sleep architecture in 1 month old infants [90]. Climatic variations may also have an impact on infant sleep development; however, more research is needed to clarify the mechanisms involved.

### Strengths and limitations of this review

The main strength of this study is the thorough follow-up of methodological advice on scoping reviews given over the last 20 years [37–40, 45]. The scope of the study was limited to advice on healthy infant sleep in the first 6 months of life. However, the selection of studies uncovered a complex and manifold package of knowledge. The authors have strived to select studies that are of high quality, recently published, and tap different important aspects involved in the facilitation of early infant sleep. Thus, previously important contributions to this field of knowledge may not be mentioned in this review. Studies focusing on how somatic health problems such as colds, itchy skin, and eating challenges may interrupt infant sleep were excluded.

## Conclusions

This scoping review documents a wide range of factors and themes that may be relevant to early preventive communication with expectant and new parents. Factors related to safe infant sleep and healthy sleep development are interwoven. Health practitioners in regions with a common health policy should search for an agreement on how to combine these factors in communicative interactions with parents. Parents want coherent and personalized services regarding infant sleep issues, and health practitioners involved in follow-up services need to cooperate in the design of appropriate programs. Different sources of information can be used by different participants or technological platforms [91]. It seems important that professionals and stakeholders within each country come together to develop a common approach about when, what, and how to communicate important sleep-related knowledge to new families.

## Data Availability

Not applicable.

## Abbreviations

AAP: American Academy of Pediatrics
CBR: Consistent bedtime routines
GP: General practitioner
JBI: Joanna Briggs Institute
P-CBS: Parent-child bedsharing
NTS: Nighttime-sleep
OSF: Open Science Framework
SBY: Sleep Baby & You (education program in UK)
SUID: Sudden unexpected infant death, here including SIDS
SV: Screen viewing
TST: Total sleep time
TNTS: Total nighttime-sleep
UK: United Kingdom
WHO: World Health Organization
